# Draft Genome Sequences of Three *Flavobacterium psychrophilum* Strains Isolated from Coldwater Disease Outbreaks at Three Production Hatcheries

**DOI:** 10.1128/genomeA.00035-16

**Published:** 2016-03-10

**Authors:** Regg Neiger, Milton Thomas, Seema Das, Michael Barnes, Brian Fletcher, Kevin Snekvik, Jim Thompson, Joy Scaria

**Affiliations:** a Department of Veterinary and Biomedical Sciences, South Dakota State University, Brookings, South Dakota, USA; b Department of Game, Fish and Parks, Pierre, South Dakota, USA; c Washington Animal Disease Diagnostic Laboratory, Pullman, Washington, USA

## Abstract

We report here the genome sequences of three *Flavobacterium psychrophilum* strains causing a bacterial coldwater disease (BCWD) outbreak, isolated from infected rainbow trout from hatcheries in Montana and South Dakota. The availability of these virulent outbreak-causing strain genome sequences will help further understand the pathogenesis of BCWD.

## GENOME ANNOUNCEMENT

Bacterial coldwater disease (BCWD) is a disease that affects a number of free-ranging and cultured salmonid and a variety of nonsalmonid fish species (1–4). BCWD typically occurs in water temperatures just above freezing to 30°C, and it is most prevalent and serious at ≤10°C. BCWD has been reported in Australia, Belgium, Canada, Chile, Denmark, France, Finland, Germany, Italy, Japan, South Korea, Spain, the United Kingdom, and the United States (2). The causative agent of BCWD is the yellow-pigment-producing bacterium *Flavobacterium psychrophilum*. The most commonly recognized symptoms of BCWD are tailrot or peduncle disease, necrotic myositis, and cephalic osteochondritis (2). BCWD has a significant economic impact on aquaculture operations in the United States and Canada. The genome sequences of the *F. psychrophilum* vaccine strain (5), type strain (6), and a European isolate are available (7). Here, we report the sequencing of three BCWD outbreak-associated *F. psychrophilum* strains isolated from rainbow trout (*Oncorhynchus mykiss*) from three different hatcheries in the United States.

For isolating genomic DNA, strains were grown in tryptone yeast extract medium for 5 days at 20°C. DNA from each strain was isolated from 1.0 ml of grown cultures using the E.Z.N.A. bacterial DNA kit (Omega Bio-tek, Norcross, GA). In accordance with the manufacturer’s protocol, sequencing libraries were prepared using 1.0 ng of genomic DNA using the Nextera XT kit (Illumina, San Diego, CA). The genomes were sequenced on an Illumina MiSeq platform using V2 paired-end chemistry (2 × 250 bp). The sequencing reads were assembled into contigs using the SPAdes genome assembler version 3.5.0. The genomes were then annotated using the NCBI Prokaryotic Genomes Annotation Pipeline (PGAP) (http://www.ncbi.nlm.nih.gov/genome/annotation_prok).

The general properties of the sequenced *F. psychrophilum* strains are shown in Table 1. The genome size and G+C content were similar to those of the previously sequenced type and reference strains of *F. psychrophilum*. Analysis revealed the presence of efflux and response elements conferring resistance to copper, lead, cadmium, and zinc in all three genomes. Since the exposure of fish to copper sulfate solution is one of the treatments used against *F. psychrophilum* infection (8), the presence of these resistance elements in these outbreak-causing strains might be conferring resistance. Genes conferring resistance to fluoroquinolones and beta-lactam antibiotics were also detected in all three genomes. The addition of antibiotics to diluents and water-hardening solutions in hatcheries is a suggested treatment for preventing the spread of BCWD (9). The presence of multiple antibiotic resistance genes in the outbreak-associated strains might have aided in the survival of these strains. The availability of these genomes of outbreak-causing strains, along with other genomes of strains published previously, could be helpful in further understanding the mechanisms behind the pathogenesis of *F. psychrophilum.*

**TABLE 1:** Metadata for *F. psychrophilum* strains isolated from coldwater disease outbreaks in U.S. hatcheries

Strain name	GenBank accession no.	Source tissue	Source state	Yr	Genome size (bp)
11754	LNTP00000000	Whole tissue homogenate	South Dakota	2011	2,945,333
17830	LNTQ00000000	Swab from sagittal section of head	South Dakota	2011	2,822,685
Pullman	LNNF00000000	Spleen	Montana	2010	2,713,260

### Nucleotide sequence accession numbers.

The accession numbers of the three *F. psychrophilum* genome sequences are listed in Table 1.
